# Effects of feeding a *Bacillus*-based probiotic to grazing Nellore bulls finished on tropical pasture

**DOI:** 10.1007/s11250-026-05430-x

**Published:** 2026-10-18

**Authors:** Rodrigo J. Oliveira, Barbara C. Afonso, Bernardo C. Petean, Giovanna L. O. Araujo, Pedro H. C. Ribeiro, Ariany F. Toledo, Danilo Domingues Millen

**Affiliations:** School of Agricultural and Veterinarian Sciences, São Paulo State University (UNESP), Jaboticabal, São Paulo 01049-010 Brazil

**Keywords:** *Bos indicus*, Fiber, Grazing, Probiotic, Rumen

## Abstract

Interest in probiotics as an alternative to ionophores has increased, particularly in pasture-based systems using energy supplementation. While these additives aim to improve feed efficiency and mitigate acidosis risk, probiotics may also modulate rumen function by enhancing fiber digestion. This study evaluated the effects of including a *Bacillus*-based probiotic in an energy supplement on intake, performance, total tract digestibility, and rumen and cecum morphometrics of finishing Nellore cattle. Twenty-eight bulls (502.6 ± 7.0 kg) were blocked by initial body weight and assigned to four paddocks of *Urochloa brizantha* cv. Marandu. A ground corn-based supplement was offered daily at 0.3% of body weight for 112 d with either no probiotic (CON; *n* = 14) or 2 g/animal of *Bacillus licheniformis* and *Bacillus subtilis* (6.4 × 10⁹ CFU/g; *n* = 14) using a Calan gate system. Forage intake and fecal output were estimated using indigestible neutral detergent fiber and titanium dioxide. Probiotic supplementation reduced pasture intake (6.32 vs. 7.18 kg/d; *P* = 0.04), total dry matter intake (8.23 vs. 9.25 kg/d; *P* = 0.02), and total crude protein intake (0.83 vs. 0.92 kg/d; *P* = 0.02), as well as NDF intake (4.40 vs. 5.04 kg/d; *P* = 0.02), without affecting nutrient digestibility (*P* > 0.10). Average daily gain was reduced (0.75 vs. 0.91 kg/d; *P* = 0.02), as was final P8 fat thickness (6.47 vs. 7.49 mm; *P* = 0.05), whereas feed efficiency (0.098 vs. 0.101; *P* = 0.76), hot carcass weight (318.61 vs. 320.32 kg; *P* = 0.70), and dressing percentage (53.88 vs. 52.65%; *P* = 0.30) were unchanged. Rumen and cecum morphometrics were not affected. Supplementation with this Bacillus-based probiotic did not improve performance of pasture-finished Nellore bulls, indicating limited benefits under these conditions and highlighting the need to clarify interactions among probiotic strains, supplementation level, and rumen kinetics.

## Introduction

Supplementation with probiotic additives has been investigated in ruminants, primarily due to their effects on the host microbiota. These additives are selected to improve rumen fermentation and potentially act as growth promoters in the beef cattle industry (Calaca et al. 2022). However, their efficacy remains variable, possibly due to differences in basal diet composition and, consequently, shifts in gastrointestinal microbial dynamics (Uyeno et al. 2015).

Probiotics are live microorganisms administered to confer health benefits in animals (Krehbiel et al. 2003). Their most significant effects have been reported during stressful periods, such as weaning, the transition to lactation, and the shift from high-forage to high-concentrate diets, improving rumen fermentation, immune function, and enhancing feed efficiency in beef and dairy cattle (Krehbiel et al. 2003; Chaucheyras-Durand and Durand 2010).

Most studies have focused on *Lactobacillus spp*., *Saccharomyces cerevisiae*, and *Bifidobacterium* (Mansilla et al. 2023; Parra et al. 2021). Recently, attention has turned to *Bacillus subtilis* and *Bacillus licheniformis*, spore-forming, Gram-positive bacteria that produce fibrolytic, amylolytic, and proteolytic enzymes (Elshaghabee et al. 2017; Su et al. 2020). These species are of growing interest for their potential to enhance nutrient digestibility through enzymatic activity and stimulation of ruminal microorganisms.

In forage-based systems, *Bacillus spp*. direct-fed microbials have been reported to reduce intake, increase total-tract digestibility of dry matter, crude protein, and fiber, and reduce plasma urea-N, suggesting improved nutrient utilization and reduced nitrogen waste (Limede et al. 2025). However, other studies have shown no improvement in digestibility, even with high-fiber diets (Calaca et al. 2022; Dias et al. 2022). This variability in response suggests that the effects of *Bacillus* may not depend solely on dietary neutral detergent fiber (**NDF**) content, but also on synergistic interactions among strains and levels that could enhance the cellulolytic capacity of the rumen microbiome (Wang et al. 2016).

According to Calaca et al. (2022), the absence of effects on performance or carcass characteristics may be due to a long adaptation period before supplementation, which promoted a stable microbial community in healthy animals, in which probiotic benefits are minimal. In addition, *Bacillus* species have been reported to reduce diarrhea and stimulate immunity in calves (Le et al. 2017; Yao et al. 2020), demonstrating an ability to grow in the intestine, which may positively impact the health and function of the entire gastrointestinal tract (Green et al. 1999; Fuerniss et al. 2022).

The probiotic inclusion rate used in the present study (2 g/animal/day; 6.4 × 10⁹ CFU/animal/day) was based on previous research evaluating the same *Bacillus*-based direct-fed microbial containing *Bacillus licheniformis* and *Bacillus subtilis* in finishing beef cattle (Cordeiro et al. 2024). Although that study was conducted under feedlot conditions, information regarding the efficacy of this supplementation strategy in tropical grazing systems with low concentrate intake remains limited. Therefore, the same probiotic dose was used as an exploratory application to evaluate its potential under grazing conditions characterized by high forage intake and low starch availability, where bacterial enzymatic activity may interact differently with the dietary substrate than under feedlot conditions.

In this context, the objective of this study was to evaluate the effects of a *Bacillus*-based probiotic on performance, apparent nutrient digestibility, carcass traits, and rumen and cecum morphometrics of Nellore bulls finished on tropical pasture consuming an energy-protein-based supplement. We hypothesized that *Bacillus* supplementation would increase forage digestibility and improve the performance of Nellore bulls during the finishing phase in the wet season.

## Materials and methods

The Ethical Committee approved all experimental procedures involving animal care and management for Animal Research of the São Paulo State University (protocol CEUA 10691663539/2026). This experiment was conducted at the Animal Beef Cattle Department, University of São Paulo, Jaboticabal, UNESP, from December 2023 until April 2024.

### Animals and treatments

Twenty-eight Nellore bulls with an initial body weight (BW) of 502.6 ± 7.0 kg from a commercial farm were enrolled in this study. Four paddocks (2.5 ha each) containing *Urochloa brizantha* ‘Marandu’ served as blocks based on forage mass. Seven animals were allocated to each paddock, and within each block, bulls were randomly assigned to one of the two experimental treatments, so that both treatments were represented within every paddock. The experimental treatments were individually offered through the energy-protein supplement using the Calan gate system, allowing the probiotic to be mixed into each animal’s supplement immediately before feeding. Thus, the individual bull was considered the experimental unit, and the paddock was included in the statistical model as a blocking factor.

The bulls remained exclusively on pasture, with ad libitum access to water and to an energy-protein-based supplement that was offered at 0.3% BW once a day for 112 d, containing the following treatments: (1) without any additive (CON; *n* = 14) or (2) same supplement containing 2 g of probiotic/animal per day (Probiotic-supplement; *n* = 14) throughout the trial. The probiotic was composed of *Bacillus licheniformis* and *Bacillus subtilis* (6.4 × 10⁹ CFU/g).

The energy-protein-based supplement was offered using a Calan gate system (American Calan, Northwood, NH). Before the start of the experiment, all bulls underwent a 14-d adaptation period to the Calan gate system to ensure that each animal was conditioned to access only its assigned feeder. During this period, grass hay was offered, and the animals were continuously monitored to confirm correct feeder use. Throughout the experimental period, supplement was offered individually through the assigned Calan gate, and intake was visually monitored daily to ensure that each bull consumed its entire allotment and to prevent access to supplements intended for other animals.

The chemical compositions of the pasture from the whole period are presented in Table 1. The experimental supplement was formulated based on the chemical analysis of pasture from the experimental paddocks, conducted 15 days prior to the start of the study. The supplement consisted of 81.2% finely-ground corn, 13.4% cottonseed meal, and 5.4% mineral premix, providing the following guaranteed levels per kg: calcium – 185 g, phosphorus – 80 g, sodium – 120 g, magnesium – 12 g, sulfur – 20 g, cobalt – 60 mg, copper – 1,350 mg, iodine – 90 mg, manganese – 1,200 mg, selenium – 20 mg, and zinc – 3,450 mg.

**Table 1:** Chemical composition of the Marandu grass pasture (*Urochloa brizantha* cv. Marandu) and supplement fed to Nellore bulls during the finishing phase

	Days during the experimental period			
Marandu grass pasture	28	56	84	112
Dry matter (DM), %	36.7	43.5	47.6	66.4
*Nutritional composition (% DM)*				
Crude Protein	6.8	7.1	7.2	4.2
Neutral Detergent Fiber	65.0	64.2	63.1	70.2
Acid Detergent Fiber	44.1	43.8	41.5	38.4
Ash	6.9	7.1	6.5	6.4
*Supplement − 0.3% body weight* ^*1*^				
DM, %	88.1			
*Nutritional composition (% DM)*				
Crude Protein	14.4			
Neutral Detergent Fiber	9.9			
Total digestible nutrients	83.9			
Ash	7.7			

^1^Supplement composed, on dry matter basis, by 81.2% finely-ground corn, 13.4% cottonseed meal, and 5.4% mineral premix (18.5% Ca, 8% P, 1.2% Mg, 12% Na, 2% S, 60 mg/kg Co, 1350 mg/kg Cu, 90 mg/kg I, 1200 mg/kg Mn, 20 mg/kg Se, and 3450 mg/kg Zn). The supplement provided to the animals maintained a consistent nutritional composition and ingredient inclusion throughout the study

The supplement was formulated in accordance with NASEM (2016) recommendations to support an average daily gain of 0.89 kg. The supplement was weighed daily on an electronic scale and administered once daily at 0800 h. All equipment used for supplement handling, including pails, bags, and shovels, was color-coded and designated exclusively for a single treatment to prevent cross-contamination.

At the beginning and end of each experimental period, the bulls were weighed after 16 h of feed withdrawal. Intermediate weightings were conducted every 28 days without fasting. The average daily gain (ADG) was calculated as the difference between the final and initial weights, divided by the number of days in each grazing cycle. Supplement, pasture, and total dry matter intake were used to calculate feed efficiency from average daily gain data. Longissimus muscle area, subcutaneous fat thickness, P8 fat thickness, and marbling were measured via ultrasound at the beginning and end of the study according to Perkins et al. (1992). Daily gains in these parameters were calculated as the difference between the two measurements divided by the number of days on feed. Images were collected using an Aloka SSD-1100 Flexus RTU unit (Aloka Co. Ltd., Tokyo, Japan) with a 17.2 cm, 3.5 MHz probe.

Blood samples were collected on days 0 and 112 directly from the coccygeal vein. Immediately after collection, samples were centrifuged, and plasma was separated and stored at − 80 °C until analysis. Plasma glucose concentrations were determined using commercial kits (LABTEST Diagnostica S.A., Lagoa Santa, MG, Brazil) and analyzed in an automatic biochemistry analyzer (Model SBA–200, CELM, Barueri, SP, Brazil). Plasma IGF-I concentrations were determined by chemiluminescence using the IMMULITE 1000 system (Siemens Healthcare Diagnostics, Los Angeles, CA, USA) with kit lot 168. The intra-assay coefficients of variation were 4.79% for level 1 and 1.98% for level 2.

### Forage production and quality

Forage samples were collected by paddock on days 0, 14, 42, 70, and 98 throughout the experimental period. Concentrate samples were collected on the same days. All samples were dried in a forced-air oven at 65 °C for 72 h to determine dry matter (DM) content. After drying, samples were ground in a Wiley mill with a 1-mm screen and analyzed in duplicate for crude protein (CP; method 984.13; AOAC International 2012), NDF (Van Soest et al. 1991), modified for use in an Ankom-200 fiber analyzer (Ankom Technology Corp., Fairport, NY), and acid detergent fiber (ADF; method 973.18; AOAC International 2012), also modified for use in an Ankom-200 fiber analyzer (Ankom Technology Corp.).

### Pasture intake and apparent digestibility of nutrients

Forage samples were obtained on days 14, 42, 70, and 98 by observing grazing behavior and collecting material that matched the quantity, leaf-stem-dead proportions, and leaf length selected by the animals (Campbell and Arnold 1973). Samples were oven-dried at 65 °C for 72 h and ground for chemical composition and indigestible NDF (iNDF) analysis.

The internal marker iNDF was used to measure pasture intake. Forage, energy-protein supplement, and feces samples were ground to pass through a 2 mm screen and analyzed for iNDF after 288 h of in situ rumen incubation. The intake of iNDF from the supplement was estimated as: Supplement iNDF intake (g/d) = Supplement intake (g/d) ×[iNDF] in supplement (g/g DM), and it was assumed to be fully excreted in feces. By subtracting the supplement iNDF intake from the total iNDF excreted in feces, the difference was assumed to be attributed to the iNDF from pasture. Then, individual pasture intake was estimated as: Pasture intake (kg/d) = (Fecal iNDF excretion (g/d) - supplement iNDF intake (g/d)) / Pasture [iNDF] (g/ kg DM).

Titanium dioxide (TiO2) was used to estimate fecal excretion. For nine consecutive days of each period (days 7–15, 35–43, 63–71, and 91–99), all bulls received 15 g TiO2 daily with the supplement. During the last five days of dosing, individual fecal samples were collected after spontaneous defecation. Fecal samples were pooled to obtain one sample per animal per period, and titanium concentration was analyzed by atomic absorption spectrophotometry as described by Myers et al. (2004). Fecal excretion (FEx; kg DM/d) was calculated as: FEx = daily TiO2 dose (g/d) / fecal [TiO2] (g/kg DM).

Apparent digestibility coefficients were calculated as: (([nutrient]pasture × pasture intake) + ([nutrient]supplement × supplement intake) − ([nutrient]feces × FEx)) / (([nutrient]pasture × pasture intake) + ([nutrient]supplement × supplement intake)).

### Slaughter and carcass yield

At the end of the trial at day 112, the bulls were slaughtered at a commercial slaughterhouse (Frigol, Lençois Paulista, São Paulo, Brazil). Hot carcasses were separated into two symmetrical sections, weighed to obtain hot carcass weight (HCW), and individually identified. Dressing percentage was calculated as the ratio of HCW to final BW for each animal (Pereira et al. 2020).

After bull evisceration, the rumen was washed and scored for the presence of lesions (rumenitis) and abnormalities (e.g., papillae clumped) using a 10-point scale (0 = no lesions and abnormalities and 10 = all rumen areas compromised by severe ulcerative lesions) as described by Bigham and McManus (1975); and Pereira et al. (2016). All rumens were scored by two trained individuals, who were blinded to the treatments. The final data represented the average of the two scores.

Additionally, a 1-cm² fragment of each rumen was collected from the dorsal cranial sac and placed in PBS for morphometric measurements, following the methodology of Resende Júnior et al. (2006). The number of papillae per square centimeter of the rumen wall (NOP) was manually determined. Twelve papillae were randomly collected from each fragment scanned, and the mean papillae area (MPA) was determined using an image analysis system (Image Tool, version 2.01 alpha 4, UTHSCSA Dental Diagnostic Science, San Antonio, TX). The rumen wall absorptive surface area (ASA) in cm² was calculated using the following equation: *ASA = 1 + (NOP × MPA) − (NOP × 0.002)*, where 1 represents the 1-cm² fragment collected, and 0.002 is the estimated basal area of papillae in square centimeters. The papillae area, expressed as a percentage of ASA, was calculated as follows: *Papillae Area (%) = (NOP × MPA) / ASA × 100.* Similarly, a 1-cm² fragment of each rumen was collected from the ventral cranial sac for histological assessment. Histological sections were stained with hematoxylin and eosin, embedded in paraffin wax, and sectioned (Odongo et al. 2006). Histological measurements, including papillae height, papillae width, papillae surface area, and keratinized layer thickness, were performed on four papillae per animal using a computer-aided light microscope image analysis.

Cecum lesions evaluation was performed after cattle evisceration, and all washed cecum were scored. Cecum epithelium was classified according to the presence of cecal wall inflammation, lesions, and petechiae using a scale of 0 (no lesions noted) to 10 (severe lesions), according to Pereira et al. (2020). All cecum were scored by two trained individuals, who were blinded to the treatments. The final data represented the average of the two scores. In addition, a 1 cm^2^ fragment was collected from the center of the cecal epithelium for histological assessment and preserved in 10% buffered paraformaldehyde until subsequent histological analysis (Devant et al. 2016). For histological analysis of cecal epithelium, tissue samples were dehydrated, embedded in paraffin wax, sectioned at 6 μm, and stained with hematoxylin and eosin. Histological measurements, including crypt depth and goblet cell density, were determined in 10% of the total number of crypts per animal using a light microscope.

### Statistical analysis

The experimental design was a complete randomized block design, with paddock as the blocking factor. The animal’s BW was homogeneous and, as a result, BW was not included as a blocking factor in the model. Data were analyzed using PROC MIXED in SAS (SAS Institute Inc. 2002). All response variables were tested for normality using the Shapiro-Wilk and Kolmogorov-Smirnov tests, and no data transformation was necessary as the residuals were normally distributed. Rumen lesion scores met the assumptions of normality and were analyzed using parametric methods. The statistical model used was: Yijk = µ + Ti + bj + eijk, where µ = was the overall mean, Ti = was the fixed effect of treatment (control or probiotic), bj = was the random effect of block, and eijk = was the residual error. Means were obtained using the LSMEANS statement. The significance level was set at *P* ≤ 0.05, and 0.05 < *P* ≤ 0.10 was considered a tendency.

## Results

The study started in the summer (rainy season) and was finished in the transition to winter (dry season), and as a result, the nutritional composition of the forage varied over the experimental period (Table 1). Dry matter content increased from 36.7% to 66.4%. Crude protein ranged from 6.8% to 7.2% until day 84, decreasing to 4.2% at day 112. The NDF remained relatively stable between 63.1% and 65.0% until day 84, after which it increased to 70.2% by day 112. On the other hand, ADF decreased slightly over time, from 44.1% to 38.4%. Ash content remained constant, ranging from 6.4% to 7.1%. The supplement was provided at 0.3% of BW throughout the experiment.

**Fig. 1:**
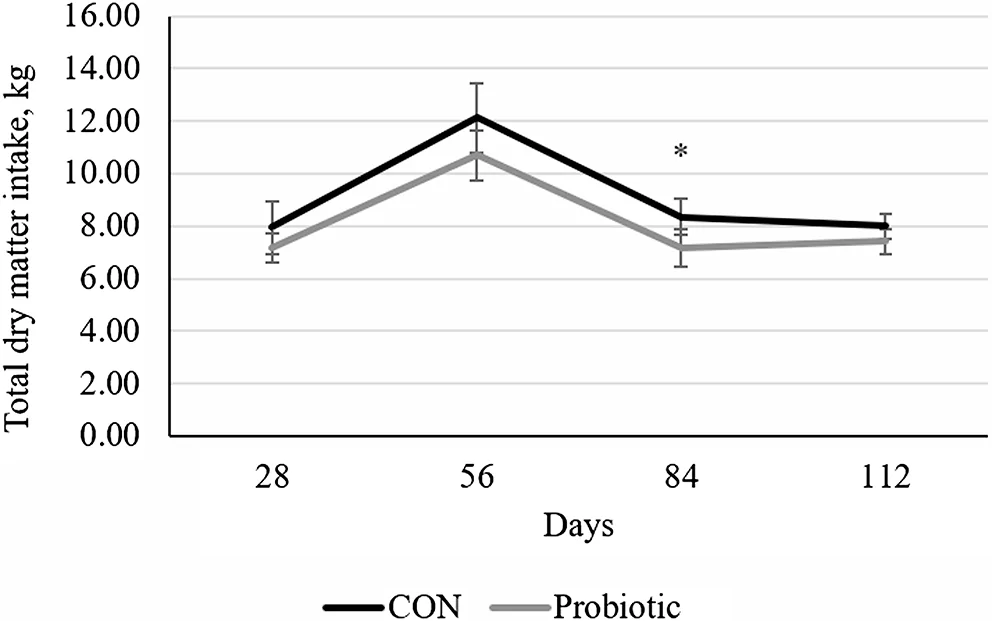
Total dry matter intake (pasture + supplement) of Nellore bulls receiving an energy-protein supplement at 0.3% of BW, without additive (CON) or with a probiotic, on days 28, 56, 84, and 112 of the experimental periods. The (*) indicates a significant difference between treatments (*P* = 0.03)

Intake results are presented in Table 2. Cattle consuming the probiotic supplement reduced overall pasture intake from 7.18 vs. 6.32 kg/d; *P* = 0.04. Supplement intake was not affected by treatments; however, total DM intake, pasture + supplement, decreased (*P* = 0.02; Fig. 1) in bulls receiving probiotic-supplement, mainly due to the lower pasture intake. This reduction was particularly evident during the 57–84-d period, coinciding with the lower pasture intake observed during this same period 6.54 vs. 5.45 kg/d; *P* = 0.05, while supplement intake remained similar between treatments.

**Table 2:** Pasture, supplement, and total dry matter intake of Nellore bulls finished on tropical pasture and fed a *Bacillus*-based probiotic

Days	Treatments*		SEM^1^	*P* – value^2^
	Control	Probiotic		Treatment
*Pasture intake*,* kg*				
0–28	6.20	5.42	0.735	0.41
29–56	10.23	8.84	1.103	0.26
57–84	6.54	5.45	0.579	0.05
85–112	6.16	5.61	0.502	0.31
Overall mean	7.18	6.32	0.698	0.04
*Supplement intake*,* kg*				
0–28	1.74	1.72	0.070	0.27
29–56	1.88	1.84	0.061	0.13
57–84	1.80	1.72	0.066	0.02
85–112	1.83	1.79	0.062	0.20
Overall mean	1.81	1.77	0.066	0.12
*Total dry matter intake*,* kg*				
0–28	7.95	7.14	0.783	0.40
29–56	12.12	10.68	1.141	0.24
57–84	8.34	7.16	0.692	0.04
85–112	7.98	7.41	0.484	0.29
Overall mean	9.25	8.23	0.795	0.02

*CON; energy-protein supplement without any feed additive (*n* = 14) - Probiotic; energy-protein supplement containing 2 g of probiotic/animal per day (*n* = 14). The probiotic was composed of *Bacillus licheniformis* and *Bacillus subtilis* (6.4 × 10⁹ CFU/g). ^1^Standard error of mean;^2^ P-value; The significance level was set at *P* ≤ 0.05, and 0.05 < *P* ≤ 0.10 was considered a tendency

Results of CP and NDF intake are presented in Tables 3 and 4. Overall, because probiotic supplementation reduced pasture intake, nutrient intake, including CP and NDF, also decreased (*P* = 0.02). Apparent nutrient digestibility data are shown in Table 5. Despite the lower pasture intake observed in bulls receiving a probiotic supplement, the digestibility of dry matter, CP, and NDF did not differ from the control group.

**Table 3:** Crude protein intake, on a dry matter basis, of Nellore bulls finished on tropical pasture and fed a *Bacillus*-based probiotic

Days	Treatments*		SEM^1^	*P* – value^2^
	Control	Probiotic		Treatment
*Crude protein intake from pasture*,* kg*				
0–28	0.64	0.55	0.08	0.39
29–56	0.99	0.86	0.11	0.27
57–84	0.56	0.46	0.05	0.05
85–112	0.44	0.42	0.09	0.72
Overall mean	0.66	0.57	0.06	0.03
*Crude protein intake from supplement*,* kg*				
0–28	0.24	0.24	0.01	0.27
29–56	0.27	0.27	0.01	0.14
57–84	0.27	0.26	0.01	0.03
85–112	0.27	0.26	0.01	0.20
Overall mean	0.26	0.26	0.01	0.09
*Total crude protein intake*,* kg*				
0–28	0.88	0.79	0.08	0.37
29–56	1.26	1.13	0.12	0.25
57–84	0.83	0.72	0.06	0.03
85–112	0.70	0.68	0.09	0.65
Overall mean	0.92	0.83	0.07	0.02

*CON; energy-protein supplement without any feed additive (*n* = 14) - Probiotic; energy-protein supplement containing 2 g of probiotic/animal per day (*n* = 14). The probiotic was composed of *Bacillus licheniformis* and *Bacillus subtilis* (6.4 × 10⁹ CFU/g). ^1^Standard error of mean;^2^ P-value; The significance level was set at *P* ≤ 0.05, and 0.05 < *P* ≤ 0.10 was considered a tendency

**Table 4:** Neutral detergent fiber intake, on a dry matter basis, of Nellore bulls finished on tropical pasture and fed a *Bacillus*-based probiotic

Days	Treatments*		SEM^1^	*P* – value^2^
	Control	Probiotic		Treatment
^*3*^*NDF intake from pasture*,* kg*				
0–28	3.89	3.39	0.466	0.41
29–56	6.19	5.38	0.623	0.27
57–84	4.40	3.70	0.387	0.05
85–112	4.20	3.69	0.372	0.15
Overall mean	4.76	4.13	0.443	**0.02**
*NDF intake from supplement*,* kg*				
0–28	0.30	0.30	0.01	0.27
29–56	0.28	0.28	0.01	0.13
57–84	0.26	0.25	0.01	0.02
85–112	0.27	0.26	0.01	0.20
Overall mean	0.28	0.27	0.01	0.13
*Total NDF intake*,* kg*				
0–28	4.19	3.69	0.470	0.40
29–56	6.47	5.66	0.632	0.26
57–84	4.67	3.95	0.394	0.05
85–112	4.34	3.98	0.331	0.31
Overall mean	5.04	4.40	0.451	**0.02**

*CON; energy-protein supplement without any additive (*n* = 14) - Probiotic; energy-protein supplement containing 2 g of probiotic/animal per day (*n* = 14). The probiotic was composed of *Bacillus licheniformis* and *Bacillus subtilis* (6.4 × 10⁹ CFU/g). ^1^Standard error of mean;^2^ P-value; The significance level was set at *P* ≤ 0.05, and 0.05 < *P* ≤ 0.10 was considered a tendency.^3^NDF; Neutral detergent fiber

**Table 5:** Dry matter and nutrient digestibility of Nellore bulls finished on tropical pasture and fed a *Bacillus*-based probiotic

Days	Treatments*		SEM^1^	*P* – value^2^
	Control	Probiotic		Treatment
*Dry matter digestibility*,* %*				
0–28	47.01	46.93	3.394	0.96
29–56	55.59	56.53	4.217	0.52
57–84	31.90	35.03	4.787	0.17
85–112	36.70	39.57	1.985	0.19
Overall mean	42.54	44.46	2.795	0.19
*Crude protein digestibility*,* %*				
0–28	50.06	49.80	2.320	0.92
29–56	47.15	46.01	4.081	0.68
57–84	33.40	35.48	3.470	0.50
85–112	30.82	33.84	4.045	0.20
Overall mean	40.45	41.68	1.295	0.39
*NDF digestibility*,* %*				
0–28	44.23	44.50	4.801	0.92
29–56	56.54	58.03	3.582	0.40
57–84	34.35	36.45	3.746	0.39
85–112	39.47	39.76	2.746	0.90
Overall mean	44.61	44.59	2.651	0.99

*CON; energy-protein supplement without any additive (*n* = 14) - Probiotic; energy-protein supplement containing 2 g of probiotic/animal per day (*n* = 14). The probiotic was composed of *Bacillus licheniformis* and *Bacillus subtilis* (6.4 × 10⁹ CFU/g). ^1^Standard error of mean;^2^ P-value; The significance level was set at *P* ≤ 0.05, and 0.05 < *P* ≤ 0.10 was considered a tendency

**Fig. 2:**
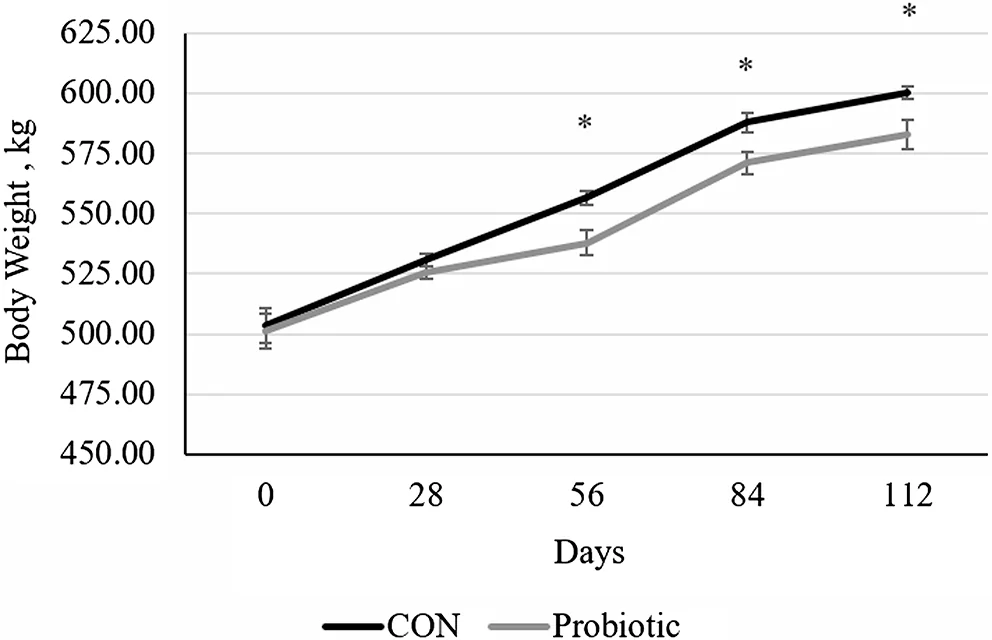
Body weight (BW) of Nellore bulls receiving an energy-protein supplement at 0.3% of BW, without additive (CON) or with a probiotic, on days 0, 28, 56, 84, and 112 of the experimental periods. The (*) indicates a significant difference between treatments (*P* < 0.05)

Performance results are presented in Table 6. Overall, the average daily gain of cattle receiving the probiotic supplement was negatively affected (*P* = 0.02), as well as the final BW (600.5 vs. 582.9 kg - *P* = 0.02; Fig. 2). The reduction in BW was already evident at day 56 and persisted at days 84 and 112, following the period in which lower pasture intake was observed. Thus, the lower BW observed at day 56 was maintained through the subsequent periods, with probiotic-supplemented bulls not recovering the difference in BW by the end of the experimental period. However, the feeding of a probiotic supplement did not affect feed efficiency (*P* > 0.10). The blood status of glucose and IGF-I was not affected by probiotic supplementation as well (*P* > 0.30).

**Table 6:** Performance and blood metabolites of Nellore bulls finished on tropical pasture and fed a *Bacillus*-based probiotic

Days	Treatments*		SEM^1^	*P* – value^2^
	Control	Probiotic		Treatment
*Body weight*,* kg*				
0	503.66	501.53	7.271	0.69
28	530.72	525.48	2.760	0.21
56	556.57	537.96	4.078	< 0.01
84	587.86	571.12	4.217	0.01
112	600.47	582.94	4.364	0.02
*Average daily gain*,* kg*				
0–28	1.11	0.92	0.098	0.21
0–56	1.01	0.68	0.062	< 0.01
0–84	1.05	0.85	0.050	0.01
0–112	0.91	0.75	0.039	0.02
28–56	0.88	0.48	0.092	0.01
56–84	1.13	1.17	0.088	0.74
84–112	0.38	0.48	0.071	0.23
*Feed efficiency*, kg/kg				
Pasture	0.129	0.128	0.0089	0.94
Supplement	0.495	0.448	0.0215	0.11
Overall	0.101	0.098	0.0058	0.76
*Blood profile*				
Glucose, mg/dL	86.82	95.06	6.646	0.41
IGF-1, ng/mL	260.83	269.63	23.031	0.79

*CON; energy-protein supplement without any additive (*n* = 14) - Probiotic; energy-protein supplement containing 2 g of probiotic/animal per day (*n* = 14). The probiotic was composed of *Bacillus licheniformis* and *Bacillus subtilis* (6.4 × 10⁹ CFU/g). ^1^Standard error of mean;^2^ P-value; The significance level was set at *P* ≤ 0.05, and 0.05 < *P* ≤ 0.10 was considered a tendency

Carcass characteristics are presented in Table 7, showing that HCW (*P* = 0.70) and dressing percentage (*P* = 0.30) were not affected by the treatments. Similarly, most ultrasound traits evaluated were not impacted by probiotic supplementation, except for final P8 fat thickness and P8 fat thickness daily gain (*P* = 0.05).

**Table 7:** Carcass characteristics of Nellore bulls finished on tropical pasture and fed a *Bacillus*-based probiotic

	Treatments*		SEM^1^	*P* – value^2^
	Control	Probiotic		Treatment
Hot carcass weight, kg	320.32	318.61	4.176	0.70
Dressing, %	52.65	53.88	0.751	0.30
*Longissimus muscle area*,* cm*^*2*^				
Initial	58.94	60.95	2.293	0.49
Final	73.07	69.13	2.408	0.10
Gain	0.117	0.082	0.022	0.10
*Subcutaneous fat thickness*,* mm*				
Initial	2.72	2.76	0.334	0.92
Final	6.27	6.02	0.322	0.59
Gain	0.031	0.029	0.0031	0.59
*P8 fat thickness*,* mm*				
Initial	3.10	3.23	0.250	0.71
Final	7.49	6.47	0.347	**0.05**
Gain	0.039	0.029	0.0031	**0.05**
*Marbling*,* %*				
Initial	3.91	4.18	0.152	0.22
Final	6.03	5.60	0.299	0.30

*CON; energy-protein supplement without any additive (*n* = 14) - Probiotic; energy-protein supplement containing 2 g of probiotic/animal per day (*n* = 14). The probiotic was composed of *Bacillus licheniformis* and *Bacillus subtilis* (6.4 × 10⁹ CFU/g). ^1^Standard error of mean;^2^ P-value; The significance level was set at *P* ≤ 0.05, and 0.05 < *P* ≤ 0.10 was considered a tendency

Finally, no changes were observed in ruminal or cecal epithelial variables of Nellore bulls consuming a probiotic supplement (Table 8). However, there was a trend (*P* = 0.08) for the feeding of a probiotic supplement to reduce the ruminal ASA.

**Table 8:** Rumen and cecum morphometrics of Nellore bulls finished on tropical pasture and fed a *Bacillus*-based probiotic

Iten	Treatments*		SEM^1^	*P* – value^2^
	CON	Probiotic		Treatment
*Lesion scores*				
Rumen	0.50	0.64	0.165	0.55
Cecum	1.57	1.85	0.420	0.66
*Rumen morphometric - macroscopic*				
Number of papillae	56.73	52.56	5.065	0.57
Papillae area, cm^2^	0.42	0.38	0.030	0.36
^ 3^ASA, cm^2^ / cm^2^ of rumen wall	29.13	21.51	3.012	0.08
Papillae area, % of ASA	96.22	95.57	0.412	0.27
*Rumen morphometric - microscopic*				
Height, mm	3.87	3.94	0.296	0.86
Width, mm	0.76	0.72	0.028	0.25
Keratin layer thickness, µm	9.19	8.34	0.389	0.13
Papillae area, mm^2^	0.98	1.08	0.066	0.27
*Cecum morphometric - microscopic*				
Crypt depth, mm	42.35	41.47	1.448	0.67
Number of enterocytes	19.46	18.82	0.809	0.58
Crypt depth: enterocyte ratio	2.20	2.25	0.091	0.72
Number of goblets cells	0.02	0.04	0.091	0.69
Number of disrupted goblet cells	0.02	0.04	0.015	0.25

*CON; energy-protein supplement without any additive (*n* = 14) - Probiotic; energy-protein supplement containing 2 g of probiotic/animal per day (*n* = 14). The probiotic was composed of *Bacillus licheniformis* and *Bacillus subtilis* (6.4 × 10⁹ CFU/g). ^1^Standard error of mean;^2^ P-value; The significance level was set at *P* ≤ 0.05, and 0.05 < *P* ≤ 0.10 was considered a tendency.^3^ ASA; absorptive surface area

## Discussion

In livestock systems, dietary manipulation is a common strategy, as ingredient availability and nutritional value vary across regions. During the finishing phase, cattle do not require specific amounts of fiber or non-fiber carbohydrates; instead, these fractions supply fermentable energy and physically effective fiber that support rumen function and meet the energy demands associated with target rates of gain (NASEM 2016). These adjustments are particularly relevant in tropical grazing systems, where forage mass and chemical composition fluctuate substantially throughout the year, as observed in the present study. Under such conditions, supplementation becomes essential to ensure an adequate nutrient supply.

Beyond diet formulation, the use of feed additives, including probiotic approaches, must be evaluated with respect to factors such as survival, thermal stability, storage stability, and resistance to gastrointestinal conditions (Bernardeau et al. 2017; Cappellozza et al. 2023). Probiotics based on *Bacillus spp*. are promising feed additives because they are supplied as spores and can grow in both the rumen and the intestine, potentially enhancing gastrointestinal health and function (Green et al. 1999; Fuerniss et al. 2022; Luise et al. 2019). In vitro studies have shown that these strains produce a wide range of enzymes (Qiao et al. 2010; van Dijl and Hecker 2013), which may enhance the degradation of fiber and starch substrates (Pan et al. 2022; Cappellozza et al. 2023).

Evidence from other probiotic strains indicates that microbial modulation can favor fibrolytic populations under specific conditions. Supplementation with *Enterococcus faecium* and *Saccharomyces cerevisiae* improved ruminal pH regulation and lactate turnover in bulls fed high-starch diets, thereby creating conditions that may favor fibrolytic species such as *Ruminococcus flavefaciens* and *Fibrobacter succinogenes* (Chiquette et al. 2012). Such mechanisms help explain why probiotic benefits are often more pronounced in diets with higher levels of non-fiber carbohydrates, in which pH fluctuations are greater and microbial modulation exerts a more substantial influence.

Although we did not measure ruminal fiber degradability, a recent study supplementing cattle with 3 g/day of a *Bacillus licheniformis* and *Bacillus subtilis* mixture (1.6 × 10⁹ CFU/g of each strain) reported increased NDF and organic matter degradability in cattle fed a forage-based diet, without affecting intake, suggesting improved microbial utilization of fiber under high-forage conditions (Eyre et al. 2025). However, this mechanism was not supported by our results, since total apparent digestibility was not affected. Therefore, the lower dry matter intake likely resulted in reduced nutrient and energy intake, which is consistent with the lower average daily gain and final body weight observed in probiotic-supplemented bulls. Because total apparent digestibility remained unchanged, the probiotic did not appear to compensate for the lower feed intake through improvements in overall digestive efficiency.

In the present study, bulls receiving 2 g/day of a *Bacillus licheniformis* and *Bacillus subtilis* mixture (6.4 × 10⁹ CFU/g) exhibited a 12.5% reduction in pasture DM intake compared with the CON treatment. Intake regulation in cattle is multifactorial and influenced by body composition, physiological state, environmental stressors, and feed additives (NASEM, 2016). Under specific conditions, reductions in intake have been associated with improved digestibility mediated by the enzymatic activity of *Bacillus spp*., especially for starch (Elshaghabee et al. 2017; Luise et al. 2019; Cappellozza et al. 2023b; Cordeiro et al. 2024). However, this mechanism was not supported under the conditions of the present study, as total apparent digestibility was unaffected by probiotic supplementation. Thus, although improved digestibility has been reported in previous studies, it does not explain the reduction in pasture and total dry matter intake observed in the present experiment. Instead, the lower nutrient intake likely resulted in lower energy intake, which was consistent with the lower average daily gain and final body weight of probiotic-supplemented bulls.

Pan et al. (2022) reported a 5.75% increase in vitro starch digestibility using the same bacterial species. Related findings indicate that *Bacillus licheniformis* hydrolyzes starch more effectively in environments with greater starch availability (Lopez et al. 2024). Consistent with this, Cordeiro et al. (2024) reported reduced fecal starch concentrations in supplemented bulls; however, unlike previous feedlot studies reporting improvements in digestibility (Cordeiro et al. 2024), cattle in the present study received only 0.3% of body weight as an energy-protein supplement while grazing tropical pasture. Consequently, the availability of rapidly fermentable substrates, particularly starch, was substantially lower than in high-concentrate diets, potentially limiting the effectiveness of the enzymatic activities produced by *Bacillus* spp. Under these conditions, the potential enzymatic effects of *Bacillus* spp. were not reflected in improved total-tract digestibility and therefore did not compensate for the reduction in dry matter intake.

We initially hypothesized that, under lower forage intake, the probiotic would improve rumen fermentation, stimulate beneficial microbial groups, and increase fiber and total-tract nutrient digestibility (Krehbiel et al. 2003; Eyre et al. 2025), thereby enhancing gain-to-feed ratio (Lopez et al. 2024; Cordeiro et al. 2024). However, total apparent digestibility remained unchanged. Therefore, the observed reduction in feed intake was not accompanied by a compensatory improvement in nutrient utilization.

This outcome indicates that probiotic supplementation altered intake regulation without improving total-tract nutrient utilization. Consequently, the combination of reduced total dry matter intake, unchanged digestibility, and lower average daily gain, final body weight and fat thickness suggests that the probiotic reduced nutrient and energy intake without increasing digestive efficiency under the conditions of the present study.

Supporting this interpretation, Qiao et al. (2010) reported that *Bacillus licheniformis* reduced ruminal ammonia concentrations, likely due to greater microbial assimilation, suggesting shifts in fiber-degrading microbial activity. In agreement, Calaca et al. (2022) observed that bulls supplemented with probiotics during the finishing phase on pasture increased the proportion of isovalerate, a branched-chain fatty acid derived from amino acid degradation, and an essential growth factor for cellulolytic bacteria, suggesting modifications in ruminal fermentation that did not translate into improved digestive efficiency or animal performance.

Previous studies have yielded varied results, depending on dietary starch concentration. Dias et al. (2024) found no improvements in intake or nutrient digestibility; however, ADG and feed efficiency tended to increase in cattle fed high-concentrate diets containing 33% starch (DM basis) and the same *Bacillus* mixture, with a lower dose: 2 g of a mixture of *Bacillus licheniformis and Bacillus subtilis* (3.2 × 10^9^ CFU/g). Likewise, Cordeiro et al. (2024) reported reduced DMI but improved gain-to-feed ratio and enhanced markers of duodenal function in cattle fed a 50% starch finishing diet ad libitum *with B. licheniformis and B. subtilis* at the same dose used in the present experiment. Similarly, Souza et al. (2026) reported that the response to supplementation with a *Bacillus*-based direct-fed microbial varied according to grazing conditions and season, reinforcing that the efficacy of these additives in grazing cattle is highly dependent on the nutritional environment. Together, these findings indicate that responses to probiotic supplementation based on *Bacillus* are not consistent across production systems and should be interpreted within the context of diet composition and management conditions.

We expected that bulls receiving 0.3% BW of the supplement would benefit from the inclusion of probiotics to support adaptation and mitigate digestive instability. However, this supplementation rate may not create ruminal or post-ruminal challenges sufficient to activate the mechanisms through which *Bacillus* strains typically exert their beneficial effects. Taken together, these findings suggest that the probiotic’s potential benefits may depend primarily on diet composition, energy density, and substrate fermentability.

Geng et al. (2016) fed feedlot bulls with different yeast preparations and reported that active dry yeast decreased 12th-rib fat thickness compared with yeast culture, while increasing circulating triglyceride and free fatty acid concentrations. These authors suggested that probiotic supplementation increased hepatic expression of genes involved in lipid oxidation and reduced lipid accumulation in subcutaneous and mesenteric adipose tissues, findings consistent with the downregulation of genes associated with lipid uptake and lipogenesis. Although Calaca et al. (2022) also attributed thinner subcutaneous fat in probiotic-fed animals to microbial influences on lipid metabolism, in the present study, it is more plausible that the lower nutrient intake reduced the availability of fermentable substrates for ruminal fermentation, potentially reducing the production of short-chain fatty acids (Van Soest 1994), thereby decreasing the availability of substrates for lipogenesis and ultimately contributing to reduced fat deposition.

Besides a tendency toward reduced ruminal absorptive surface area (*P* = 0.08), probiotic supplementation did not affect rumen lesion scores, papillae morphometry, cecal lesion scores, or cecal histological characteristics. The absence of morphological adaptations is consistent with the lack of changes in nutrient digestibility. It indicates that the probiotic did not substantially alter gastrointestinal development under the conditions of this study. Previous studies have reported that *Bacillus* probiotic strains can reduce diarrhea and stimulate immunity in calves by downregulating oxidative processes, activating genes associated with immune function, and increasing serum alkaline phosphatase activity, which is considered a marker of improved gut immunity (Le et al. 2017; Yao et al. 2020).

A stable microbiota helps prevent gastrointestinal disorders and impaired nutrient absorption (Virgínio Júnior and Bittar 2021). In high-starch diets, the accumulation of short-chain fatty acids can compromise cecal health. Therefore, we expected that probiotic supplementation, even at 0.3% BW, would reduce cecal lesions and goblet cell disruption, both of which are indicative of cecal acidosis in ruminants. Nevertheless, the probiotic did not affect cecal parameters, possibly because the bulls were less challenged in the grazing system.

Collectively, the present findings and recent studies (Moriel et al. 2025; Souza et al. 2026) suggest that the productive responses to *Bacillus*-based direct-fed microbials are context-dependent and influenced by animal category, production system, and nutritional management. Therefore, recommendations for the use of these additives should consider the feeding strategy under which they are applied. Under the conditions evaluated in the present study, supplementation with this *Bacillus*-based probiotic at 2 g/animal/day did not provide benefits to Nellore bulls finished on tropical pasture and receiving 0.3% BW of an energy-protein supplement, as it reduced pasture intake and animal performance without improving nutrient digestibility or carcass characteristics. These findings should be interpreted specifically within the dietary and management conditions evaluated in this study. Furthermore, although the animals were the experimental unit, only four paddocks were used as blocks, which may be a limitation to address in future studies.

## Data Availability

The data supporting the findings of this study are available from the corresponding author upon reasonable request.
